# Modulation of the functional connectome in major depressive disorder by ketamine therapy

**DOI:** 10.1017/S0033291720004560

**Published:** 2022-10

**Authors:** Ashish K. Sahib, Joana R. Loureiro, Megha Vasavada, Cole Anderson, Antoni Kubicki, Benjamin Wade, Shantanu H. Joshi, Roger P. Woods, Eliza Congdon, Randall Espinoza, Katherine L. Narr

**Affiliations:** 1 Department of Neurology, Ahmanson-Lovelace Brain Mapping Center, University of California Los Angeles, Los Angeles, CA, USA; 2 Department of Psychiatry and Biobehavioral Sciences, University of California Los Angeles, Los Angeles, CA, USA

**Keywords:** Functional connectivity, ketamine, major depression, salience network

## Abstract

**Background:**

Subanesthetic ketamine infusion therapy can produce fast-acting antidepressant effects in patients with major depression. How single and repeated ketamine treatment modulates the whole-brain functional connectome to affect clinical outcomes remains uncharacterized.

**Methods:**

Data-driven whole brain functional connectivity (FC) analysis was used to identify the functional connections modified by ketamine treatment in patients with major depressive disorder (MDD). MDD patients (*N* = 61, mean age = 38, 19 women) completed baseline resting-state (RS) functional magnetic resonance imaging and depression symptom scales. Of these patients, *n* = 48 and *n* = 51, completed the same assessments 24 h after receiving one and four 0.5 mg/kg intravenous ketamine infusions. Healthy controls (HC) (*n* = 40, 24 women) completed baseline assessments with no intervention. Analysis of RS FC addressed effects of diagnosis, time, and remitter status.

**Results:**

Significant differences (*p* < 0.05, corrected) in RS FC were observed between HC and MDD at baseline in the somatomotor network and between association and default mode networks. These disruptions in FC in MDD patients trended toward control patterns with ketamine treatment. Furthermore, following serial ketamine infusions, significant decreases in FC were observed between the cerebellum and salience network (SN) (*p* < 0.05, corrected). Patient remitters showed increased FC between the cerebellum and the striatum prior to treatment that decreased following treatment, whereas non-remitters showed the opposite pattern.

**Conclusion:**

Results support that ketamine treatment leads to neurofunctional plasticity between distinct neural networks that are shown as disrupted in MDD patients. Cortico-striatal-cerebellar loops that encompass the SN could be a potential biomarker for ketamine treatment.

## Introduction

Pharmacotherapies remain the first line of treatment for major depressive disorder (MDD). However, these medications can take several weeks or longer to be effective (Rush et al., 2006) and a third of patients, defined as having treatment resistant depression, will remain refractory to two or more treatment trials (Gaynes et al., 2009; Nemeroff, 2007). Over the past decade, ketamine has been identified as a rapidly acting antidepressant (Murrough et al., 2015; Zarate et al., 2006), with robust therapeutic effects occurring within a day of a single infusion in patients with MDD (aan het Rot et al., 2010; Mathew et al., 2010; Murrough et al., 2013). Ketamine is thought to exert its antidepressant effect through *N*-methyl-d-aspartate (NMDA) receptor antagonism (Berman et al., 2000; Zarate et al., 2013) *α*-amino-3-hydroxy-5-methyl-4-isoxazolepropionic acid modulation, and/or via involvement of opiate receptors or other neurotransmitter systems (Kraus et al., 2019; Kubicki et al., 2019). Accordingly, the molecular and cellular mechanisms of ketamine's antidepressant action remain under investigation. Furthermore, since most existing studies of the systems-level neurofunctional effects of ketamine (Evans et al., 2018; Reed et al., 2018) have examined single infusion only, whether repeated administration of ketamine engages the same or additional functional brain networks is not known.

MDD is characterized by dysfunctional social, emotional, and reward-based processing, with resultant disruptions in mood, anhedonia (reduced capacity to experience pleasure), and impaired cognitive functioning (Diener et al., 2012; Gotlib & Joormann, 2010). Resting state (RS) networks using functional magnetic resonance imaging (fMRI) have been well studied in MDD (Greicius et al., 2007; Kaiser, Andrews-Hanna, Wager, & Pizzagalli, 2015). Prior research has largely focused on the default mode network (DMN), which is most active at rest and is involved in self-referential processing and rumination (Hamilton, Farmer, Fogelman, and Gotlib, 2015). However, disturbances in other networks such as salience and cognitive control networks are also reported and proposed to explain depressive symptomology in MDD (Hamilton et al., 2015; Murrough et al., 2015). Studies that have investigated RS-fMRI changes 24 h post ketamine infusion in MDD have also shown changes in salience and executive control network, suggesting that ketamine normalizes MDD-related dysfunction in these networks (Evans et al., 2018; Reed et al., 2018). However, of note, earlier RS-fMRI studies of ketamine have primarily employed seed-based approaches, which only consider a subset of functional brain networks (Anand, Li, Wang, Gardner, & Lowe, 2007; Posner et al., 2013). Other studies have adopted atlas-based approaches to identify altered networks at the whole brain network level (Fornito, Zalesky, & Breakspear, 2015; Korgaonkar, Goldstein-Piekarski, Fornito, & Williams, 2019), though this approach is also limited to examining changes in *a priori* determined atlas-based networks.

Brain networks are generally characterized by a set of brain regions (nodes) and the connections (edges) that link them (Rubinov & Sporns, 2011). Compared to *a priori* focused seed-based or atlas-based single-network analysis, whole brain RS-fMRI-based connectomics (Cole, Smith, & Beckmann, 2010; Smith et al., 2013) allow investigation of systems-level treatment effects in depression from the perspective of large-scale brain networks and their complex interactions. Such network modeling can be carried out by parcellating the RS-fMRI into a number of distinct brain regions (nodes) using high-dimensional independent component analysis (ICA), and subsequently estimating the functional connectivity (FC) (i.e. edges) in terms of temporal correlations (Chan, Park, Savalia, Petersen, & Wig, 2014; Sala-Llonch, Bartres-Faz, & Junque, 2015; Smith et al., 2015). The advantage of this method is that it does not require an *a priori* hypothesis; instead it uses all the information within the data to drive its network estimations. Thus, perturbations in functional networks or the functional connectome relating to antidepressant treatment are rendered more objective and comprehensive. In the current study, we thus used a data-driven approach to investigate the functional connectome of the brain at rest after acute (24 h after first infusion of ketamine) and serial (24 h after receiving four infusions) ketamine treatment in MDD patients. To establish whether treatment-related FC changes normalize toward control values, we also compared FC between controls and MDD patients at baseline. Though employing different methodological approaches, based on previous findings (Abdallah et al., 2017; Evans et al., 2018), we hypothesized that the network dysfunction in MDD patients would be normalized after ketamine treatment, particularly across the DMN, somato-motor network (SMN), salience network (SN) and fronto-parietal network (FPN). In addition, we aimed to identify correlates of response for MDD patients who reached remission after serial ketamine infusion and hypothesized that the FC changes (Evans et al., 2018) in the SN would help characterize response.

## Methods and materials

### Subjects

**Fig. 1.**
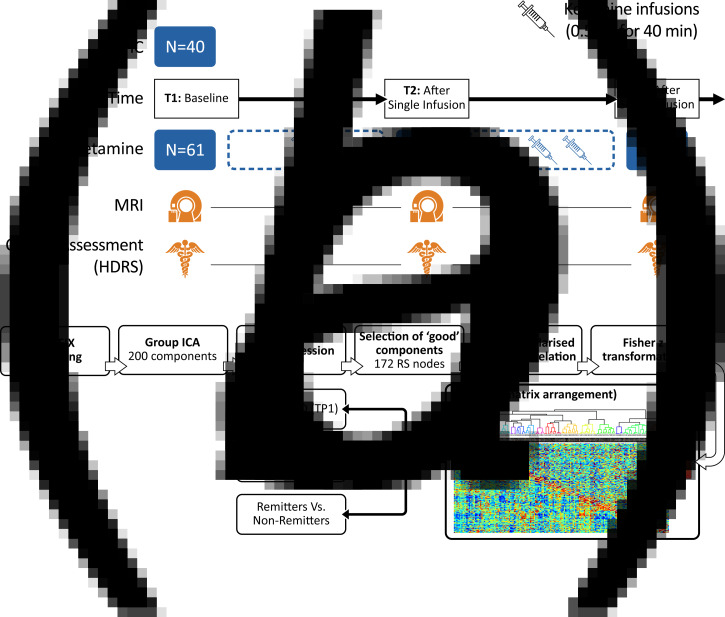
(*a*) Study design illustrating the timing of MRI sessions and clinical assessments relative to ketamine infusions. (*b*) Processing pipeline to generate the RS functional connectome. High-dimensional group-ICA and network modeling were performed using FSL-MELODIC and FSLNETS tools. *z* statistics for the full correlation (below the diagonal) and partial correlation (above the diagonal) were computed for the 172 identified nodes. The nodes were reordered according to a hierarchical clustering of the full correlation matrix. The transformed partial correlations were then arranged in the form of a network matrix (netmat), which was used to perform three main analysis: cross-sectional comparisons of HC and MDD at baseline (TP1), longitudinal comparisons of change between baseline and end of serial treatment in patients (TP1 *v.* TP3), and comparison of changes between treatment remitters *v*. non-remitters.

Participants included 40 healthy controls (HC) and 61 DSM-5 defined [SCID (First, Williams, Karg, & Spitzer, 2015)] individuals with MDD, who met criteria for treatment resistant depression [i.e. failed two adequate antidepressant trials of adequate dose and duration and had been continuously depressed for ≥6 months in the current episode (Gaynes et al., 2020), all 20–64 years of age]. Subjects were recruited from the Los Angeles area through advertisements, clinician referral, or clinicaltrials.gov (NCT02165449) and received clinical and behavioral assessments and an MRI scan. Patients received a series of four ketamine treatments over a period of 2–3 weeks. Assessments were made at three different time points: (1) initial baseline (TP1) occurring within 1 week of the first treatment (*N* = 61); (2) 24 h after the first ketamine infusion (TP2, *N* = 48) and; 24–72 h after the last ketamine infusion (TP3, *N* = 51) (Fig. 1(*a*)). Slightly fewer patients received T2 assessments due to limitations in resources for scanning after the first infusion. At each time point, depression severity was assessed using the Hamilton Depression Rating Scale (HDRS), 17-item (First et al., 2015; Hamilton, 1960). Patients whose HDRS score reached ≤7 at the end of treatment (TP3) were considered remitters (Frank et al., 1991; Zimmerman, Chelminski, & Posternak, 2004), whereas the remainder of patients completing four infusions were defined as non-remitters. Demographic and clinical information for all subjects is provided in Table 1.

**Table 1.** Patient demographic and clinical information

	HC mean (s.d.)	MDD mean (s.d.)	*T*/χ	*p*
Number of subjects (*N*)	40	61		
Gender (% female)	60	47.54	χ = 1.5	0.22
Age (years)	32.87 (12.7)	38.96 (10.7)	*T* = 2.58	0.01
Education (years)	10.71 (1.8)	10.24 (2.2)	*T* = −1.1	0.18
Duration lifetime illness (years)	N/A	22.81 (12.43)	N/A	N/A
Current episode (years)	N/A	5.5 (5.95)	N/A	N/A
Comorbid disorders^a^				
Generalized anxiety	N/A	39	N/A	N/A
Mood disorder	N/A	1	N/A	N/A
Manic episodes	N/A	1	N/A	N/A
Feeding and eating disorders	N/A	7	N/A	N/A
Substance use disorder^b^	N/A	25	N/A	N/A
Trauma and stressor-related disorders	N/A	19	N/A	N/A
ADHD	N/A	1	N/A	N/A
Somatic symptom and related disorders	N/A	1	N/A	N/A
HDRS (TP1)	N/A	19.29 (4.8)	N/A	N/A
HDRS (TP2)	N/A	12.64 (4.5)	N/A	N/A
HDRS (TP3)	N/A	8.62 (4.6)	N/A	N/A

HDRS, Hamilton Depression Rating Scale; HC, healthy controls; patients with major depressive disorder (MDD) at baseline (TP1); 24 h after the first infusion (TP2) and after fourth infusion (TP3) that included 51 participants.

a Comorbid disorders based on SCID -V.

b Substance use disorder includes several classes of substances including alcohol; caffeine; cannabis; hallucinogens; inhalants; opioids; sedatives, hypnotics or anxiolytics; stimulants; tobacco; and other or unknown substances. However, current substance abuse or dependence (ascertained by laboratory testing) or substance abuse history within the preceding 3-months were exclusionary.

Exclusion criteria for all participants included any unstable medical or neurological condition, current substance abuse, or dependence (ascertained by laboratory testing) or substance abuse history within the preceding 3-months, current or past history of psychosis, schizophrenia, mental retardation, or other developmental disorder, diagnosis of dementia and any contraindication to scanning (e.g. metal implants or claustrophobia). Exclusion criteria for HC also encompassed any current of past psychiatric condition. Prior to treatment, all patients had moderate to severe depressive symptoms as per the HDRS (score ≥17) (Hamilton, 1960). Patients were also screened to ensure no prior psychotic reactions to medications, alcoholic, or illicit substances in the past, and for other physical or clinical contraindications to ketamine. All subjects provided written informed consent following procedures approved by the University of California, Los Angeles (UCLA) Institutional Review Board (IRB).

### Ketamine treatment

Patients receiving ketamine treatment were permitted to remain on approved monoaminergic antidepressant therapy (if unchanged in the preceding 6-weeks) for the duration of the study. Benzodiazepines were discontinued the night before and morning of all study visits (e.g. scan sessions and ketamine infusion session). Patients received infusions 2–3 times a week for a total of four infusions. At each session, performed as an outpatient procedure, a single sub-anesthetic dose (0.5 mg/kg) of ketamine diluted in 60 cm^3^ normal saline was delivered intravenously via pump over a 40-min period in a private room at the UCLA Clinical Research Center or Resnick Neuropsychiatric Hospital. Vital sign monitoring included blood pressure, pulse oximetry, and respiratory rate recording every 3 min and a continuous cardiac rhythm strip. Mental status monitoring also occurred during ketamine infusion to assess for any untoward behavioral or psychological effects.

### MRI data acquisition

A Siemens 3T Prisma MRI system at UCLA's Brain Mapping Center using a 32-channel phased array head coil was used to acquire imaging data. Image acquisition sequences were identical to those used by the Human Connectome Project (HCP) Lifespan studies for Aging and Development (https://www.humanconnectome.org). The structural scans consisted of a *T*1-weighed (T1w) multi-echo MPRAGE [voxel size (VS) = 0.8 mm isotropic; repetition time (TR) = 2500 ms; echo time (TE) = 1.81:1.79:7.18 ms; inversion time (TI) = 1000 ms; flip angle (34) = 8.0°; acquisition time (TA) = 8:22 min) and a *T*2-weighted (T2w) acquisition (VS = 0.8 mm isotropic; TR = 3200 ms; TE = 564 ms; TA = 6:35 min], both with real-time motion correction (Tisdall et al., 2012). RS-fMRI data (participants were instructed to focus on a fixation cross) were acquired using two runs of a multiband EPI sequence (Ugurbil et al., 2013) with opposite phase encoding directions [VS = 2 mm isotropic; TR = 800 ms; TE = 37 ms, FA = 52°, MB accl. factor = 8; phase enc. direction = AP (run1)/PA (run2); TA = 6:41 min (per run)].

### MRI data analysis

Anatomical and functional data were visually inspected and minimally preprocessed using the HCP minimal preprocessing pipeline (Glasser et al., 2013; Smith et al., 2013) implemented using the BIDS-App (Gorgolewski et al., 2017). Structural and functional artifacts were then removed using a modified sICA + FIX [spatial ICA followed by ICA-based X-noiseifier (Griffanti et al., 2014; Salimi-Khorshidi et al., 2014)] that utilized concatenation across runs and phase encoding directions of all the fMRI data within a single scanning session (Glasser et al., 2018). MSMALL alignment was performed to identify and align corresponding cortical locations across subjects based on a combination of cortical folding, thickness, myelination, and RS FC measures (Glasser et al., 2016; Robinson et al., 2014; Robinson et al., 2018). Images with artifacts remaining after pre-processing, which comprised of one subject not counted in the reported sample size, were excluded from the study. Following preprocessing (Fig. 1(*b*)), high-dimensional group-ICA (Beckmann & Smith, 2004; Kiviniemi et al., 2009) was performed to generate a set of nodes or parcels. All the RS-fMRI scans were fed into group-level ICA to parcellate the data into a set of 200 spatially-independent components. These spatial maps were projected onto each subject's RS-fMRI time series data to derive one time series per ICA component per subject [dual regression stage 1 (Filippini et al., 2009)]. After rejecting the group-ICA components based on artifactual noise (i.e. relating either to scanning artifacts, or to non-neuronal biophysical processes such as cardiac fluctuations and head motion), 172 components remained (online Supplementary Fig. 1). For each subject, the time series associated with these (*D* = 172) components were used as nodes to perform network analysis (Smith, 2012). A *D* × *D* matrix (netmat) of connectivity estimates was generated using the FSLNets toolbox (http://fsl.fmrib.ox.ac.uk/fsl/fslwiki/FSLNets). Network modeling was carried out using partial temporal correlation between node time series. To improve the stability of the connectivity estimates L2 regularization (*ρ* = 0.1) was applied (Smith et al., 2011). Network matrix or netmat values were converted from Pearson correlation scores (*r* values) to *z* statistics using Fisher's transformation. Partial correlation *z* statistic network matrices were estimated separately for each RS-fMRI run, and then averaged across the runs for each subject, resulting in a single netmat for each subject. The 172 nodes were reordered according to hierarchical clustering of the group-average full correlation netmat using Ward's method implemented in Matlab to generate the functional connectome (Fig. 1(*b*)). Each subject's partial correlation netmat was unwrapped into a single row and combined across subjects to create a Subject × Edges matrix.

### Statistical analysis

The Subject × Edges matrix was used to evaluate group-level analyses including, examination of cross-sectional effects between diagnostic groups at baseline, longitudinal effects of ketamine treatment, and associations between change in neural response occurring after single or serial ketamine treatment and antidepressant response. A two-sample *t* test with age and sex as regressors of no interest compared cross-sectional differences in netmat between HC and MDD at baseline. To test for longitudinal effects of ketamine treatment, paired *t* tests compared netmats between time points examined pairwise, evaluating effects of both single (TP1–TP2, *n* = 48) and serial ketamine treatment (TP1–TP3, *n* = 51). Using a two-sample *t* test, we also evaluated differences in FC between remitters and non-remitters at baseline (TP1), and the change in FC (ΔFC) after end of treatment (TP1–TP3) with age and sex as regressors of no interest. The FSL randomize tool with 5000 permutations was used for multiple comparisons (controlling family-wise error, FWE) across all edges. Results that were significant at FWE-corrected *p* < 0.05 are reported. The network edges that survived statistical significance (FWE *p* < 0.05) were used in a post-hoc analysis using IBM Statistical Packages for the Social Sciences (SPSS v25) to examine relationship between variations in baseline and change in FC with percent change in clinical scores after serial ketamine (TP1–TP3/TP1). Furthermore, FC values were used as dependent measures to confirm the presence of time-by-remission status interactions employing a general linear mixed model with time (TP1 and TP3) and remitter status (remitter, non-remitter) as fixed factors. A *p* value of <0.05 was used to establish statistical significance in these post-hoc analyses. To test the longitudinal stability of the FC estimates we also compared the FC in a subsample of HC scanned at two time points over a period of 2 weeks (online Supplementary material).

## Results

### Demographic and clinical results

Sex did not differ significantly between HC and MDD groups at baseline, but patients were on average ~6 years older, *p* = 0.01 (Table 1). To control the variance associated with both sex and age, these variables were included as covariates of no interest in the cross-sectional analysis of FC comparing diagnostic groups. In MDD patients, HDRS (*F*_(1.89, 91.81)_ = 83.77, *p* < 0.0001) scores showed significant improvement across time (Table 1), and maximum improvement occurred after serial ketamine infusion (TP3). Of the 51 MDD patients who completed serial ketamine infusions, 24 (47%) achieved remission (HDRS < 7).

### Cross-sectional effects between HC and MDD at baseline

**Fig. 2.**
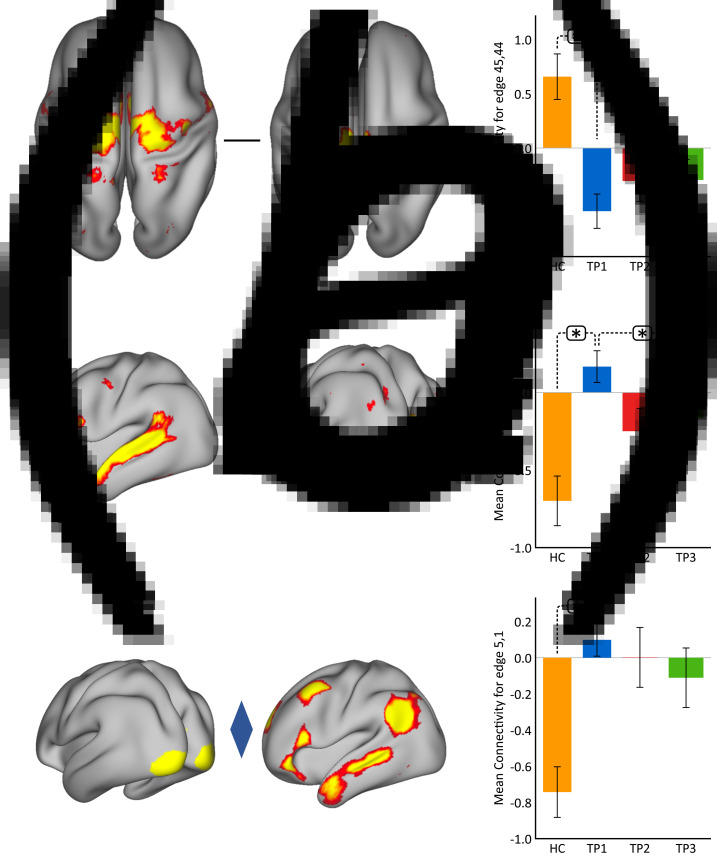
Cross-sectional effects: controls *v*. MDD. (*a*) FC between nodes in the SMN is significantly lower in MDD as compared to controls; node 45: premotor cortex, Brodmann area (BA) 6; node 44: primary motor cortex, BA 4a. (*b*) FC between association and visual network is significantly higher in MDD as compared to controls; node 25: temporal lobe; node 14: visual cortex V1 BA 17. (*c*) FC between the visual and DMN is significantly higher in MDD as compared to controls; node 5: visual cortex (V1, V2, V3); node 1: right DMN. The color of the diamond connecting the two nodes represents the sign of the group average (patients + HC at TP1) partial correlation (orange: positive, blue: negative). All images are thresholded at *z* > 8 for visualization. Bar plots show the mean FC for each of the significant (*p* < 0.05, FWE-corrected across the netmat) networks for HC and MDD patients at TP1, TP2 and TP3 (*p < 0.05).

Using *p* < 0.05 FWE correction and including age and sex as covariates, FC within the somato-motor (nodes 45 and 44) network was found significantly greater in HC as compared to MDD at baseline (Fig. 2(*a*)). Mean FC between these nodes at each time point represented in the bar plots indicates that FC in patients trends toward normalization over time with ketamine treatment. In contrast, FC between the ventral attention node (node 25) and the visual node (node 14) was significantly higher in MDD as compared to controls at TP1. As illustrated in the bar plots in Fig. 2*b*, FC between these nodes significantly reduced with ketamine treatment. Similarly, we also observed higher FC between visual cortex (node 5, encompassing areas V1, V2, and V3) and the DMN node (node 1) in MDD at TP1 that decreased with treatment (Fig. 2(*c*)). There was no significant difference in in-scanner motion metrics at the cross-sectional level as well as over time in MDD subjects (online Supplementary Fig. 2).

### Longitudinal effects of ketamine treatment on the functional connectome

**Fig. 3.**
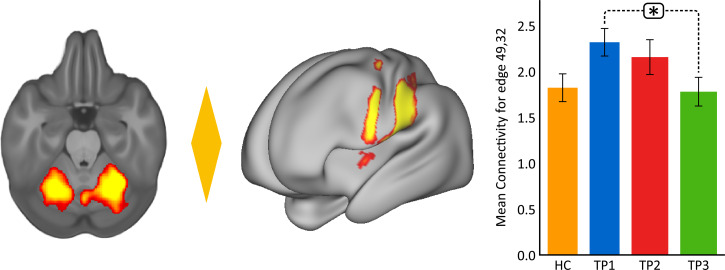
Effect of ketamine treatment after fourth infusion (TP1 *v*. TP3). The FC between the cerebellum and the SN was positively correlated and significantly reduced with ketamine treatment; node 49: cerebellar lobule VI; node 32: secondary somatosensory cortex and insula. The color of the diamond connecting the two nodes represents the sign of the group average (patients + HC at TP1) partial correlation (orange: positive, blue: negative). All images are thresholded at *z* > 8 for visualization. Bar plots show the mean FC between these nodes for HC and MDD at TP1, TP2 and TP3 (*p < 0.05).

The paired *t* test comparing the whole-brain FC at baseline (TP1) and after the fourth infusion (TP3) showed significant decrease in FC between a node in the cerebellum (node 49) and a node in the SN (node 32) at *p* < 0.05 FWE correction (Fig. 3). The bar plots show that the average FC between the cerebellum node and the SN node is much higher in MDD patients at baseline (TP1) as compared to controls. Furthermore, this increase in mean FC decreases after the first as well as the fourth infusion of ketamine. No significant differences were found in FC for the paired *t* test comparing the netmat between TP1 and TP2.

### FC changes and clinical outcome

**Fig. 4.**
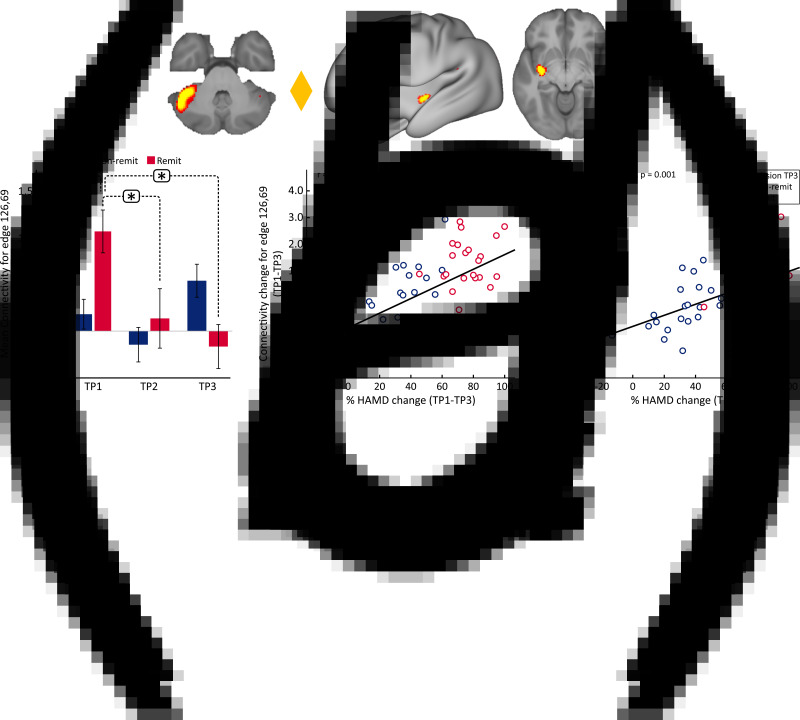
Distinct RS connections representing the difference between change in FC (TP1–TP3) for remitters and non-remitters. (*a*) The FC between the cerebellum node and the node in the striatum along with insula showed the largest change in FC between remitters and non-remitters: node 69: cerebellar left crus 1; node 126: left putamen and insula. The color of the diamond connecting the two nodes represents the sign of the group average (patients + HC at TP1) partial correlation (orange: positive, blue: negative). All images are thresholded at *z* > 8 for visualization. (b) Bar plots show the mean FC between these nodes for HC, remitters and non-remitters at TP1, TP2 and TP3 (*p < 0.05). (*c*) ΔFC (TP1–TP3) between the cerebellum and striatum showed a significant positive relationship with %HDRS change (TP1–TP3). (*d*) Baseline (TP1) FC between the cerebellum and the striatum showed a significant positive relationship with %HDRS change (TP1–TP3).

At the whole brain level, ΔFC pre-to-post treatment (T1–T3) between remitters and non-remitters showed a significant difference at *p* < 0.05 FWE correction between a node in the cerebellum (node 126) and a node in the striatum (node 69) (Fig. 4*a*). No significant differences were observed between remitters and non-remitters at baseline using *p* < 0.05 FWE correction. However, there was significant time-by-remission (*F*_(1.969)_ = 3.742, *p* = 0.027) status interaction of FC between node 126 and 69. The bar plots in Fig. 4*b* show the average FC reduces over time for remitters, whereas FC for this edge exhibits the opposite trend for non-remitters. When examining average FC for this edge in post-hoc analysis, results also show significantly higher FC for remitters at baseline (TP1) as compared to HC (Fig. 4*b*). In addition, there was a significant positive correlation between ΔFC at the end of treatment and %HDRS change (TP1–TP3) after the fourth infusion. Finally, there was a significant positive relationship between FC of the cerebellum node and the SN node at baseline (TP1) with change in %HDRS (TP1–TP3) after the fourth infusion.

## Discussion

Sub-anesthetic doses of ketamine delivered in either its racemic form of *S*(*+*) and *R*(−) enantiomers (Murrough et al., 2013; Zarate et al., 2013), or as (*S*)*-*ketamine only (Daly et al., 2018; Singh et al., 2016), can reduce depressive symptoms within hours. However, relatively little is known about its effects on brain function at the systems-level following single or repeated ketamine therapy. Furthermore, MDD patients can vary in their response to ketamine treatment with remission rates varying between 29% and 44% (Wilkinson et al., 2017; Zarate et al., 2006). Whether patterns of FC can predict or elucidate the neurofunctional mechanisms associated with remission following ketamine therapy remains mostly unaddressed. Moreover, previous FC studies in MDD have to date exclusively focused on investigating specific brain networks such that other changes in the functional connectome associated with ketamine may be missed. The current study thus leveraged a data-driven network modeling approach to investigate how single and repeated ketamine administration modulates this functional connectome in MDD. To this end, 172 distinct functional nodes were generated from a high-dimensionality group-ICA that map several large-scale networks including auditory, somatosensory, motor, and visual activity as well as higher cognitive processes like executive function (Suri et al., 2017). Using these nodes/networks, distinct RS connections were mapped to generate a functional connectome. Changes in the functional connectome across time in patients, and between controls and MDD patients prior to ketamine infusion were subsequently addressed.

At the cross-sectional level, results revealed that FC within the SMN was positively correlated for controls as compared to MDD patients, where it was anticorrelated. This anticorrelation in the SMN decreased in MDD patients with ketamine treatment. Furthermore, we also showed that the FC between the VN and FPN/association network was positively correlated in MDD patients, whereas it was anticorrelated in HC. These positive correlations between the FPN/association network and VN decreased in MDD patients with ketamine treatment. These findings suggest that the disrupted network connections that we observe in MDD patients at baseline are reaching normative (control) values with ketamine treatment. Specifically, the distinct RS connection that involved the cerebellum and the SN was uniquely coupled with repeated ketamine administration. The FC between these nodes was positively correlated at baseline in MDD and much greater than HC. With repeated ketamine infusion the FC in this network decreased, again trending toward normative values. In terms of clinical response remitters showed increased positive correlation at baseline between the cerebellum and the striatum that significantly decreased with ketamine treatment. But the non-remitters showed an opposite trend that involved the FC to be similar that of controls and increased in strength at the end of ketamine treatment. Overall, these findings suggest that repeated low-dose ketamine treatment normalizes the dysfunction of the SN in MDD. In particular, the cortico-striatal-cerebellar (CSC) loops that encompass the SN could be a potential biomarker for ketamine treatment.

### Cross-sectional effects: HC *v*. MDD

Patients with MDD are known to show reduced FC within and across several brain networks at rest (Yan et al., 2019). Consistent with previous findings, MDD patients at baseline showed lower mean FC in the SMN as compared to controls. This reduced FC within the SMN may be linked with psychomotor retardation, which has been characterized as a key feature of patients with major depression (Buyukdura, McClintock, & Croarkin, 2011; Iwabuchi et al., 2015). A recent study also showed decreased FC in the SMN prior to treatment in MDD, which increased with standard pharmacotherapeutic treatment (Korgaonkar et al., 2019). Although not significant, in the current study we observed a similar effect of FC within this SMN in MDD patients with ketamine treatment. These results suggest that ketamine tends to normalize the dysfunction in this SMN that may be associated with its antidepressant effect. Furthermore, the FC between the VN and the DMN/association network was anticorrelated in controls, whereas MDD patients showed an opposite effect. Prior studies have reported disrupted FC within the VN and the DMN/association networks (Kaiser et al., 2015; Yan et al., 2019). Here, for the first time we present disrupted network connectivity across these networks as well. In addition, ketamine is known to modulate metabolism in the occipital cortex (Carlson et al., 2013), and visual cortex activity has been shown to correlate with antidepressant response (Furey et al., 2013). Notably, we have also previously found cerebral blood perfusion changes in primary and secondary visual areas following ketamine therapy to be associated with antidepressant response in an overlapping sample of patients (Sahib et al., 2020). Similarly, at the end of ketamine treatment we observed a significant modulation of FC between VN and the association network (Fig. 2*b*) with treatment. These changes showed a trend toward controls, indicating the antidepressant effects of ketamine include restoration of FC.

### Ketamine modulation of the functional connectome in MDD

Relative to baseline, 24 h after the fourth infusion of ketamine we observed a significant decrease in FC (Fig. 3) between the node in the dorsal salience (node 32) network (Abdallah et al., 2019) and the node in the SMN part of the cerebellum (node 49, Habas et al., 2009). The cerebellum participates in motor as well as non-motor functions (Habas et al., 2009) and MDD pathophysiology affects distinct sub-regions of the cerebellum that communicate with cortical networks, thereby sub-serving several cognitive and mood functions (Depping, Schmitgen, Kubera, & Wolf, 2018). Furthermore, cerebellar NMDA receptors play a vital role in motor coordination and motor learning (Sanchez-Perez, Llansola, Cauli, & Felipo, 2005) and prior studies have shown that NMDA receptor antagonists restore motor activity in mutant nice (Umemori et al., 2003).

Patients with MDD are known to have abnormal functioning of the SN (Kaiser et al., 2015; Sheline, Price, Yan, & Mintun, 2010). The SN plays a critical role in switching between task negative network (DMN) and the task positive (FPN) (Menon & Uddin, 2010). As a result, any abnormalities in the SN can affect several cognitive functions (Koechlin & Summerfield, 2007; Miller & Cohen, 2001). In particular, the region (Fig. 3) reported in this study that encompasses the SN is implicated in patients with post-traumatic stress disorder (Abdallah et al., 2019). Notably, the dysfunctions associated with the SN in MDD are also found to be normalized with ketamine treatment in prior studies (Abdallah et al., 2017; Evans et al., 2018). Although not significant, the mean FC between the node in the cerebellum and the SN in patients with MDD was greater as compared to HC. In accordance with previous ketamine studies (Abdallah et al., 2017; Evans et al., 2018), we show that there are disruptions within as well as across the SMN and SN in patients with MDD. This disruption across the SMN and the SN normalizes toward controls (Fig. 3) with repeated ketamine therapy further suggesting that neuroplasticity across these networks contributes to the antidepressant effects of ketamine at the brain systems level. A recent study that employed a data-driven approach of global brain connectivity (GBC) has also shown that early GBC in the SMN predicts response to sertraline treatment (Nemati et al., 2020). Furthermore, a recent study from our group (Sahib 2020) has also shown that functional plasticity in the SMN after ketamine treatment relates to improvements in depressive symptoms, suggesting modulation of this network plays an important role in therapeutic response.

### FC associated with treatment response

Almost half of the patients included in the current study remitted at the end of treatment. Our MDD cohort overall had a significant higher FC of the SN at baseline (Fig. 3). However, on splitting the group based on clinical outcome, only remitters showed a significantly higher FC between the cerebellum node and a node in the striatum. The basal ganglia are essential for reward-based learning (Watabe-Uchida, Eshel, & Uchida, 2017) and imaging studies have also indicated brain regions outside the basal ganglia, including the cerebellum, to be associated with reward processing (Garrison, Erdeniz, & Done, 2013; Tobler, O'Doherty J, Dolan, & Schultz, 2006). Furthermore, lesions in the cerebellum are associated with impairments in reinforcement-learning (Thoma, Bellebaum, Koch, Schwarz, & Daum, 2008). It should also be noted that the cerebellum and the basal ganglia are interconnected at the sub-cortical level (Bostan, Dum, & Strick, 2010; Haines & Dietrichs, 1984; Haines, Dietrichs, Mihailoff, & McDonald, 1997) and increased activity is observed in this circuit across various neurological disorders (Bostan & Strick, 2018). This CSC loop along with the insula has been shown to be a key neural circuit of the SN (Habas et al., 2009). In line with these findings, compared to controls, remitters in the current study showed significantly higher FC in the CSC loop as compared to controls at baseline, which significantly decreased with ketamine treatment. In contrast, non-remitters showed lower FC as compared to controls that increased with ketamine treatment. In addition, we observed a significant relationship between change in FC in this CSC circuit with improvement in clinical outcome, and baseline FC in this loop could predict end of treatment outcome. Taken together and in line with previous findings, these observations suggest that SN dysfunction in patients with MDD, particularly those more prone to reward-based deficits (CSC loops) might respond better to ketamine treatment and further studies of basal ganglia–cerebellar–cerebral cortical networks could help better understand the pathophysiology of this disorder.

### Limitations

Several limitations should be acknowledged for the current investigation. First, the current study was not a randomized clinical trial and did not include an active control condition. A previous study has implicated RS in predicting placebo effects (Sikora et al., 2016). However, it is important to note that the focus of this mechanistic clinical trial was to investigate the perturbation of the functional connectome at rest associated with ketamine rather than to address clinical efficacy, and patients serve as their own controls when examining change in FC over time. Furthermore, changes in FC over the course of ketamine treatment related to antidepressant response suggest that these changes are not reflective of placebo effects. Also, a subsample of HC subjects was scanned twice to estimate normative variance in FC over time (online Supplementary material). Notably, none of the edges that were significantly modulated with ketamine treatment showed a change in FC for HC over time in the direction of ketamine treatment (online Supplementary Fig. 3). These results are in line with other studies that demonstrate the patterns of RS brain activity measures are relatively stable across individual control subjects scanned multiple times (Almgren et al., 2018; Chen et al., 2008). Though cross-sectional changes between MDD and HC subjects were investigated to assist with the interpretation of ketamine findings, it is possible that the inclusion of patients with comorbid psychiatric diagnoses as well as the heterogeneity of symptoms among depressed individuals may have influenced the observed results between diagnostic groups. Finally, patient participants were allowed to continue concurrent stable anti-depressant medication, which may have impacted findings.

## Conclusion

The use of data-driven functional connectomics to identify brain networks at rest is a major methodological advancement that could translate to improved strategies for diagnosis and for tailoring treatment in patients with MDD. Using this approach our findings provide novel insights into the specific components of large-scale brain networks associated with ketamine therapy. We have identified disrupted FC within the SMN, and between the visual and association networks in MDD patients that normalize with ketamine treatment. Furthermore, we found distinct changes in RS FC in SN networks with respect to ketamine treatment. Finally, in terms of remission, we found greater than normal FC in the CSC loop in patients who remit, which may be a prerequisite mechanism for ketamine treatment. Future studies may expand upon the current findings, including addressing how treatment-related changes in the functional connectome vary in relation to longer term clinical outcomes, and maintenance of therapeutic response.

## Supplementary material

For supplementary material accompanying this paper visit https://doi.org/10.1017/S0033291720004560. click here to view supplementary material
