# Supplementary material for: Modulation of the functional connectome in major depressive disorder by ketamine therapy
Source: Psychol Med. 2020 Dec 3;52(13):2596–605. doi: 10.1017/S0033291720004560 (PMC9647551; doi:10.1017/S0033291720004560)
Supplement: Supplementary file 1 [file S0033291720004560sup001.zip › S0033291720004560sup007.docx]

**Supplementary Figure 1:**  Based on the independent component analysis (ICA) parcellation, the172 components used to generate the functional connectome.


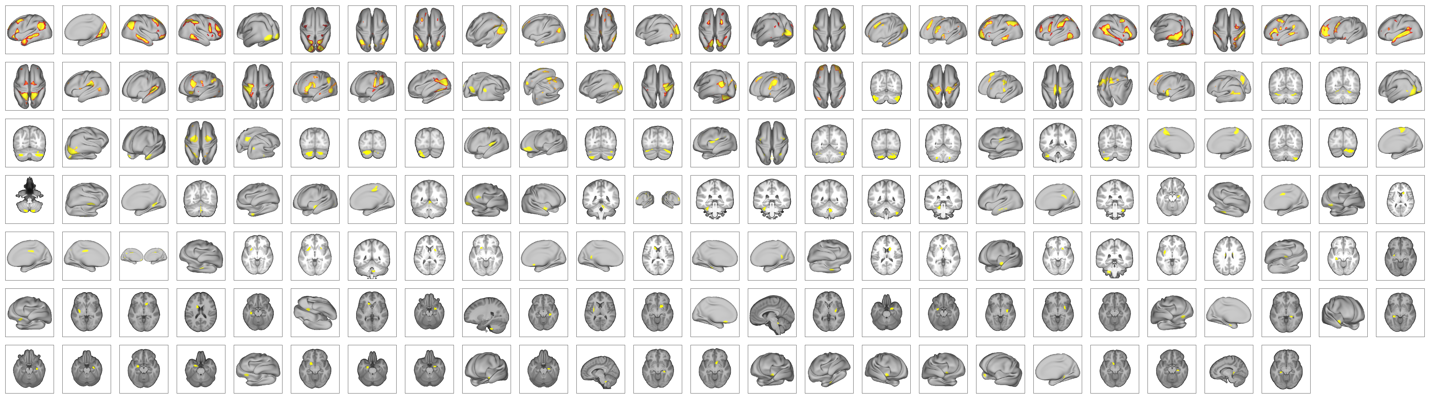


**Supplementary Figure 2:** Absolute mean of the movement parameters for controls (HC in blue at TP1 and TP2) and patients (in red at TP1, TP2 and TP3).

**Supplementary Figure 3:** Test-retest reliability of functional connectivity (FC) measures in healthy controls.

To test the stability of the FC measures over time, we acquired resting-state fMRI data in two sessions over a period of two weeks using identical image acquisition parameters as described in the methods section from 17 healthy controls (HC, mean age = 28.23±6.8, 9 females). No significant (p<0.05, FWE corrected) FC differences were found for the paired t-test comparing the netmat for HC at TP1 and TP2. The bar plots show the mean FC strength between the nodes that are reported in the study. In the post-hoc analysis, one edge (FC between DMN and primary visual cortex) that was not significantly modulated with ketamine treatment showed a significant change over a period of two weeks in HC. This change in FC in the visual cortex may be attributed to the resting-sate design as the subjects were instructed to focus on a fixation cross during the scan. However, none of the edges that were significantly modulated with ketamine treatment showed a change in FC for HC over time in the direction of ketamine treatment.
