# Supplementary figures and images for: Modulation of the functional connectome in major depressive disorder by ketamine therapy
Source: Psychol Med. 2020 Dec 3;52(13):2596–605. doi: 10.1017/S0033291720004560 (PMC9647551; doi:10.1017/S0033291720004560)

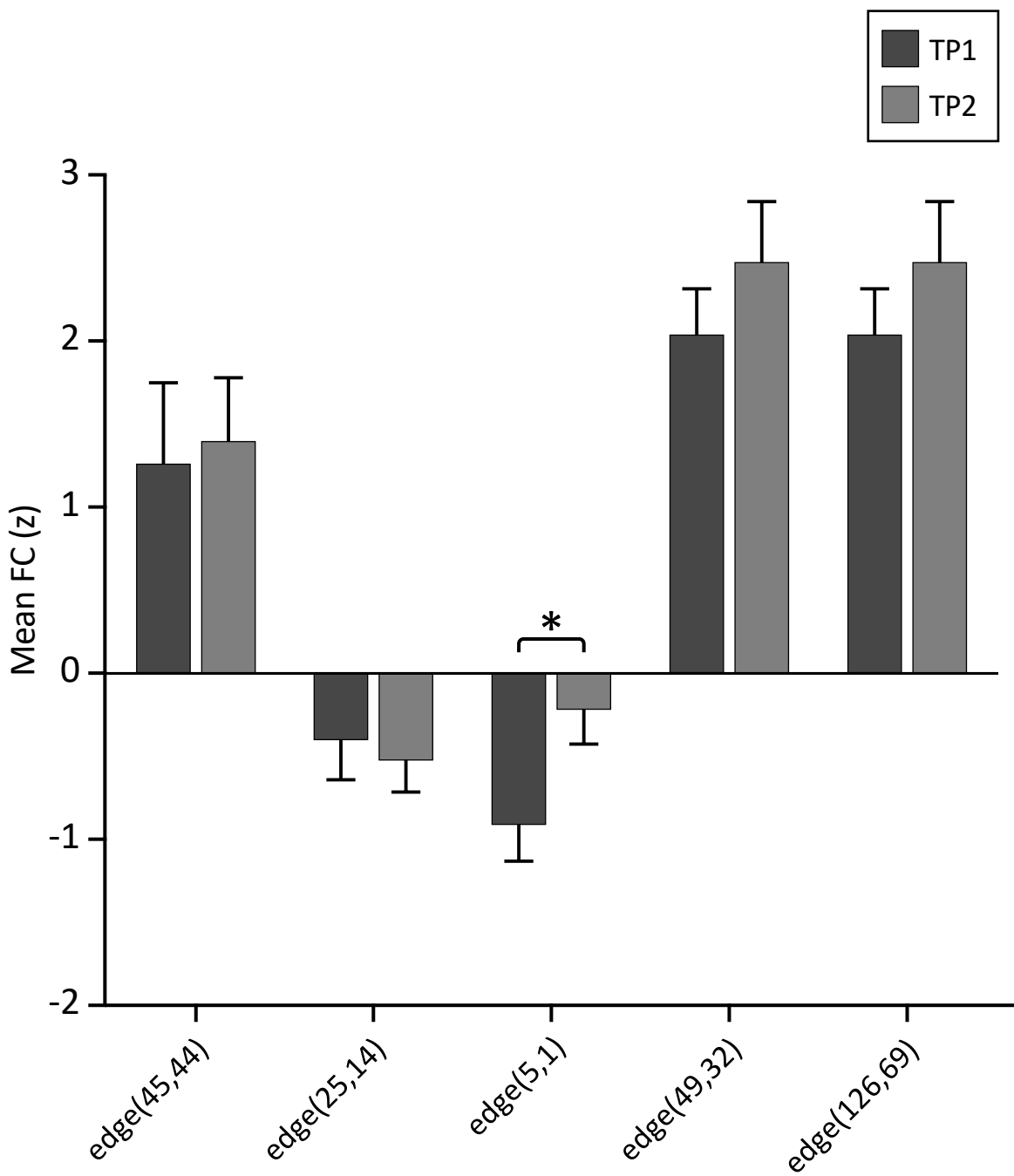

Supplement: Supplementary file 1 [file S0033291720004560sup001.zip › S0033291720004560sup001.pdf]

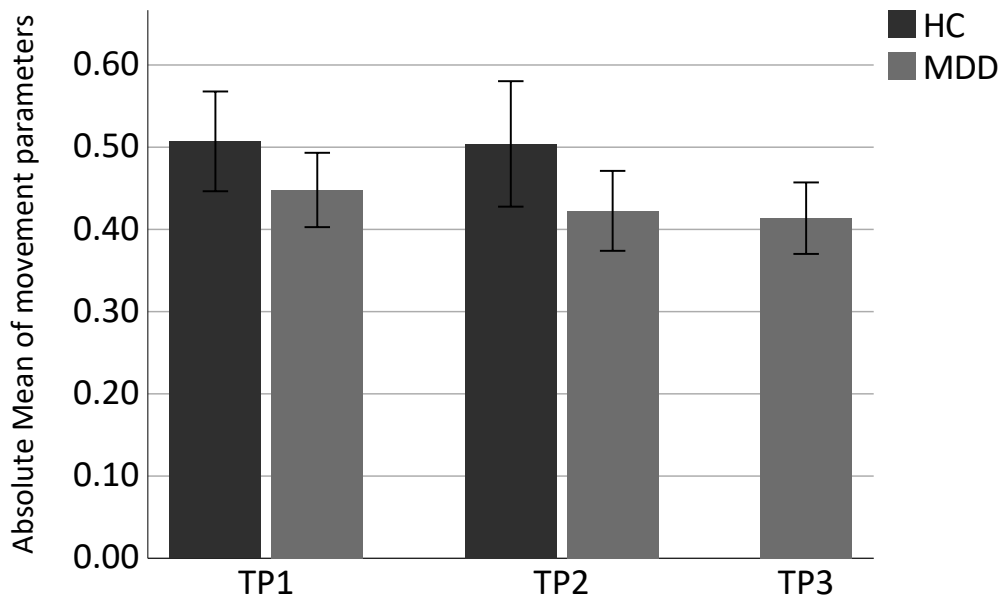

Supplement: Supplementary file 1 [file S0033291720004560sup001.zip › S0033291720004560sup002.pdf]

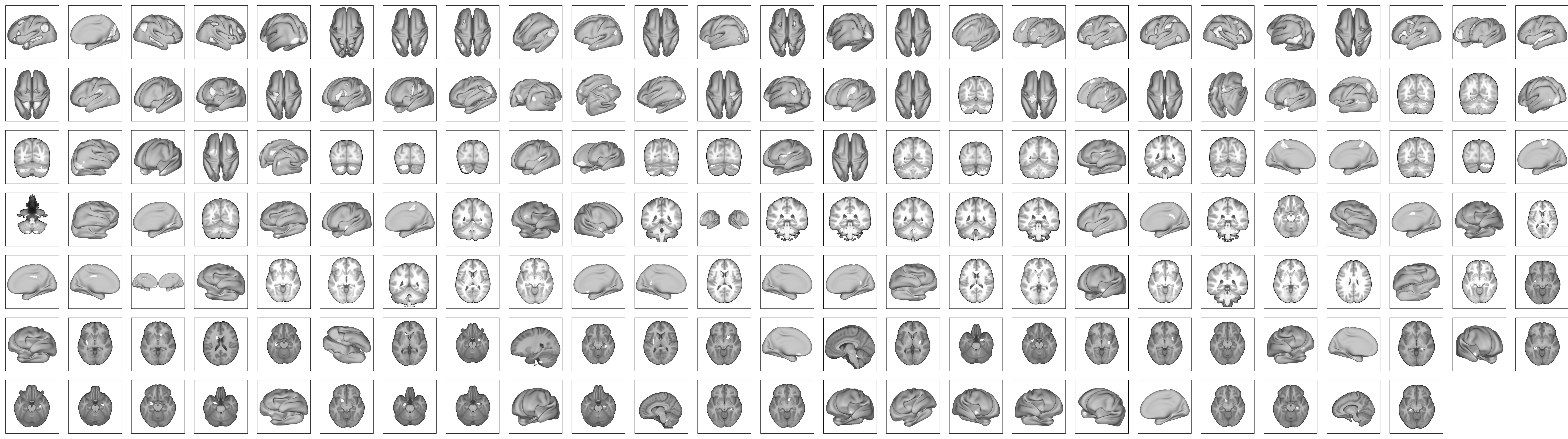

Supplement: Supplementary file 1 [file S0033291720004560sup001.zip › S0033291720004560sup003.pdf]

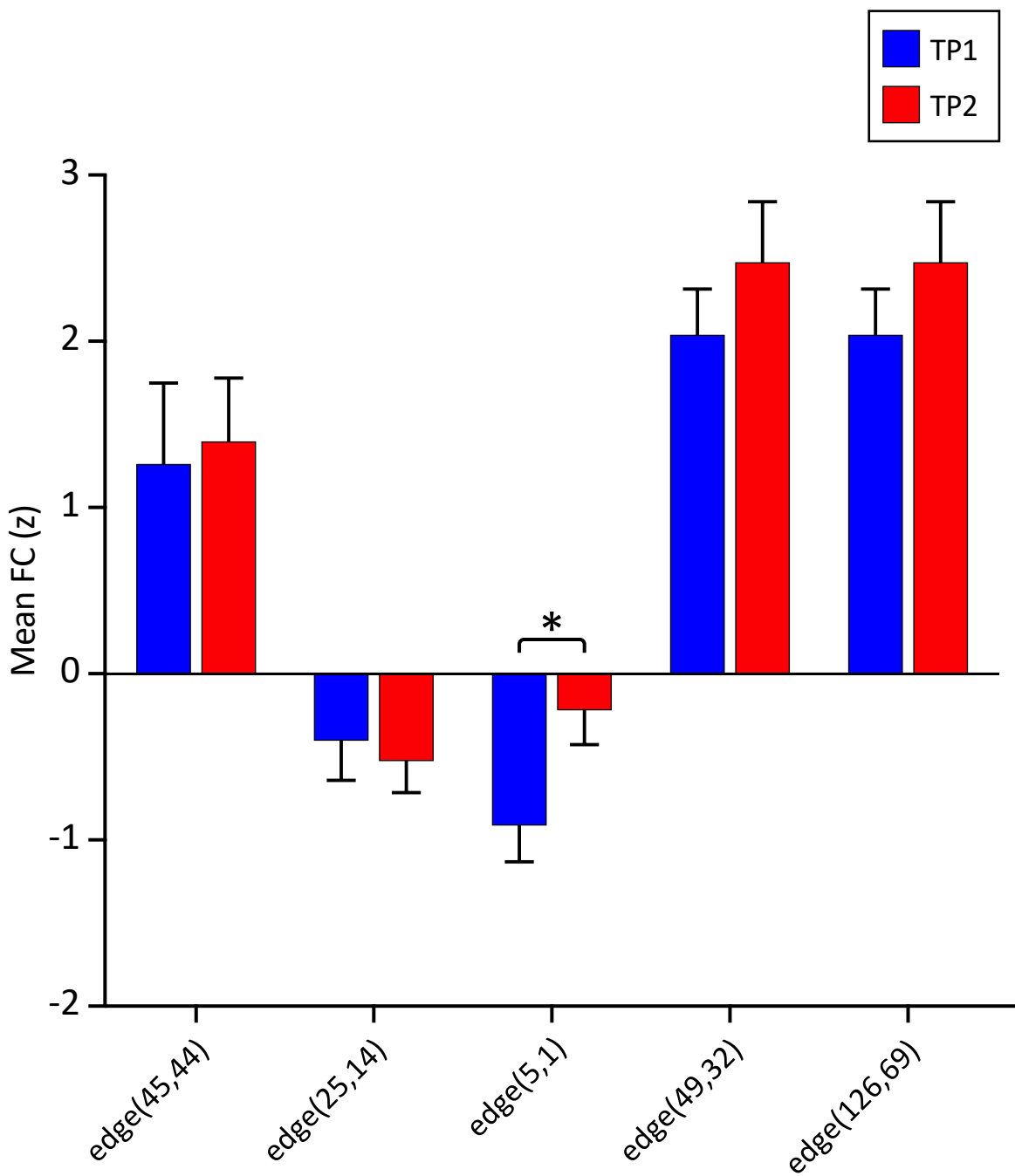

Supplement: Supplementary file 1 [file S0033291720004560sup001.zip › S0033291720004560sup004.pdf]

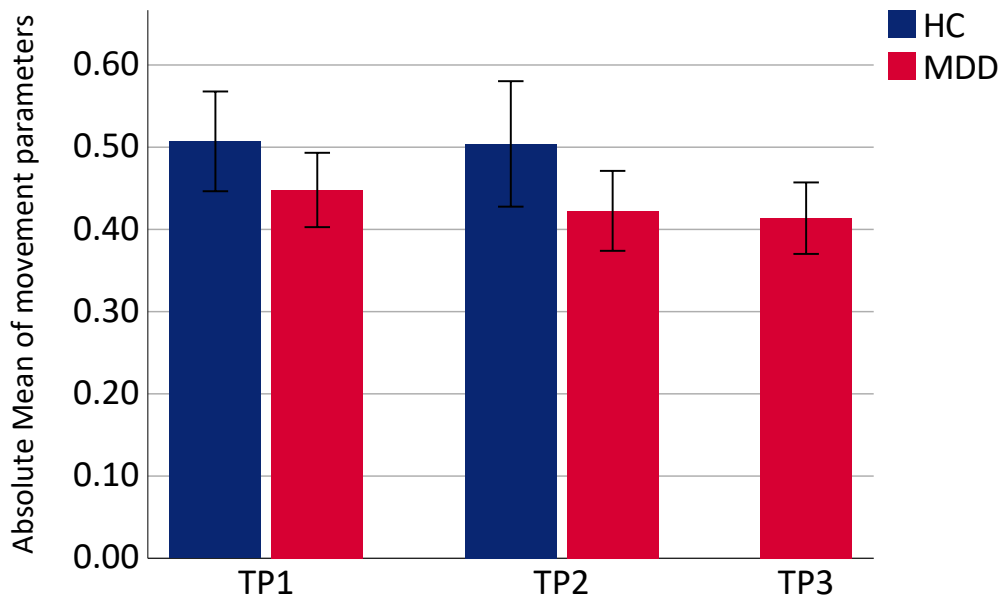

Supplement: Supplementary file 1 [file S0033291720004560sup001.zip › S0033291720004560sup005.pdf]

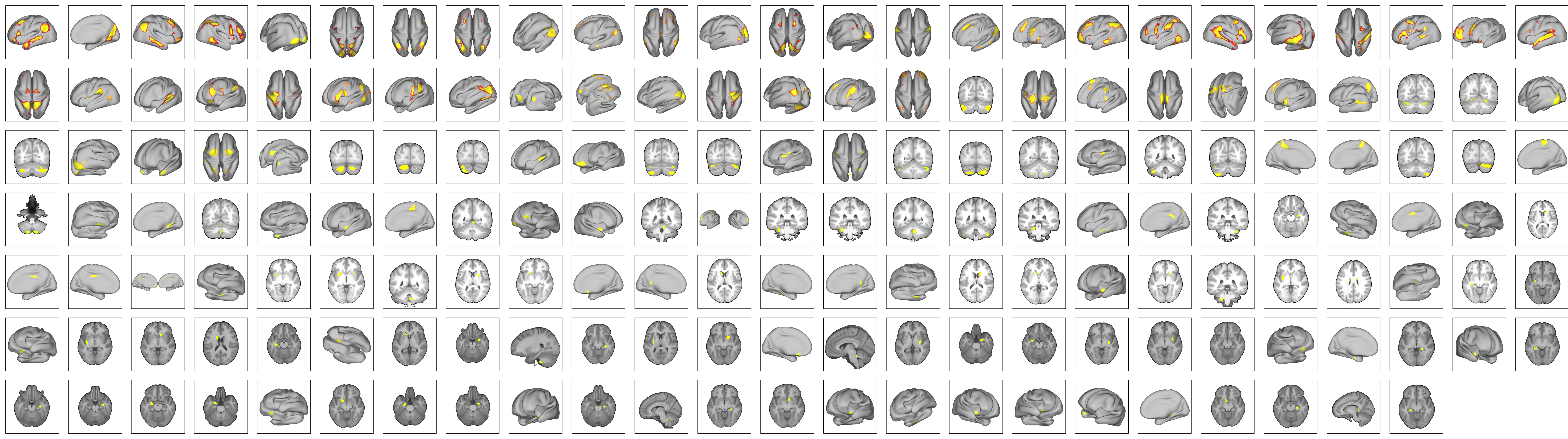

Supplement: Supplementary file 1 [file S0033291720004560sup001.zip › S0033291720004560sup006.pdf]
